# Factors affecting the acculturation strategies of unaccompanied refugee minors in Germany

**DOI:** 10.3389/fpsyg.2023.1149437

**Published:** 2023-06-19

**Authors:** Maike Garbade, Jenny Eglinsky, Heinz Kindler, Rita Rosner, Cedric Sachser, Elisa Pfeiffer

**Affiliations:** ^1^Department of Child and Adolescent Psychiatry/Psychotherapy, Ulm University, Ulm, Germany; ^2^German Youth Institute, Munich, Germany; ^3^Department of Psychology, Catholic University Eichstätt-Ingolstadt, Eichstätt, Germany

**Keywords:** unaccompanied refugee minors, acculturation, daily stressors, integration, trauma

## Abstract

**Background:**

Different acculturation strategies might be related to different mental health outcomes and social participation of unaccompanied refugee minors (URMs), but little is known about which factors influence this acculturation process. Therefore, the aim of this investigation was to examine the impact of individual, stress-related, and contextual factors on the acculturation process of URMs in Germany.

**Methods:**

A sample of *N* = 132 URMs living in child and youth welfare service facilities in Germany completed questionnaires about their acculturation orientation, traumatic experiences, daily stressors, asylum stress, and perceived social support between June 2020 and October 2021. This investigation is part of the multi-center randomized control trial BETTER CARE. Data were analyzed descriptively and via multiple hierarchical regression.

**Results:**

Integration (43.5%) and Assimilation (37.1%) were the most common acculturation strategies used by URMs. Multiple hierarchical regression models showed that daily stressors (e.g., the lack of money) were associated with a stronger orientation toward the home country, whereas traumatic events were associated with a weaker orientation toward their home country. No significant predictors were found for the orientation toward the host country.

**Discussion:**

Overall, URMs in Germany showed favorable acculturation strategies. Nevertheless, daily stressors and traumatic experiences might influence this process. The implications for practitioners and policymakers are discussed with a view to further improving the acculturation process of URMs in Germany.

**Clinical Trial Registration:** German Clinical Trials Register, DRKS00017453 https://drks.de/search/de/trial/DRKS00017453. Registered on December 11, 2019.

## 1. Introduction

As a consequence of ongoing wars, conflicts, violence or persecution, the number of refugees worldwide continues to rise. In 2020, the UNHCR estimated that 42% of all displaced persons were minors (United Nations High Commissioner for Refugees, 2021). They are often unaccompanied and are, therefore, frequently placed in child and youth welfare service (CYWS) facilities or foster care (Karpenstein and Rohleder, 2022). At the end of 2021, 17.947 unaccompanied refugee minors (URMs) and young refugee adults under the age of 21 were living in CYWS facilities in Germany (Karpenstein and Rohleder, 2022). When resettling to a new country, they have to adapt to a new life, being confronted with acculturation tasks. This entailed learning a new language, making new friends, or becoming accustomed to different traditions (Brook and Ottemöller, 2020).

Acculturation can be understood as a multidimensional, dynamic process of simultaneously adopting aspects of the receiving country and maintaining aspects of the home country (Schwartz et al., 2010). Thus, acculturation is a relevant topic for every individual, who is in contact with more than one country, such as refugees. The most cited acculturation model by Berry (1997) distinguishes between four main acculturation strategies: assimilation, marginalization, integration, and separation. Assimilation describes a strategy in which members of a minority group do not maintain their home cultural identity and are strongly oriented toward the host country. This means, that acculturating individuals might reject traditions from their home country and want to speak the language of the host country only. They often prefer to have friends from the host country than having friends from their home country (d’Abreu et al., 2019). In contrast, the Separation strategy describes the wish of the individuals to maintain their cultural identity, whereas there is no orientation toward the host country. In this case, acculturating individuals, are avoiding the participation in the host society and focus more on their origin and traditions (El-Awad et al., 2021). When individuals are oriented toward both their home and their host country, the term used is Integration. In this case, the acculturating individual practice not only traditions and languages from their home country and aims to have contact to ethnic peers, but they also aim to learn the language of the host country, try to understand new rules and traditions, and want to have friends from both, their home country and the host country (El-Awad et al., 2021). Finally, Marginalization describes the strategy adopted by individuals who are oriented neither toward their home country nor toward the host country. Individuals are not interested neither in their home country nor in the host country. Often, they are not involved in neither activities of the host society nor of their home country.

Regarding the distribution of preferred acculturation styles among refugees, Copoc (2019) has shown that the majority of adult Syrian refugees in Germany showed either the strategy of Integration (48.4%) or Assimilation (42.4%). As the acculturation process is always influenced by the attitudes of the receiving society (Berry, 1997), a similar distribution of displayed acculturation strategies could be expected for URMs in Germany. But since URMs have to deal simultaneously with both acculturation tasks and developmental tasks (Berry et al., 2006), these results cannot be generalized. It is, therefore, even more surprising that information of the preferred acculturation strategies for the specific group of URMs has been lacking up to now.

Although the acculturation model by Berry (1997) is the most common model used in this field of research, more recently this approach has been questioned because of conceptual and statistical problems. To distinguish between the four acculturation strategies proposed by Berry (1997), continuous scores of the orientation toward home and host countries are often dichotomized using median- or mean-splits. This not only leads to a loss of variance but also to smaller sample sizes in the respective groups (Maehler and Shajek, 2016). Therefore, more recently, a continuous approach is preferred, in which the orientation toward the home country and orientation toward the host country are analyzed independently (Demes and Geeraert, 2014).

Although the importance of acculturation for the development of URMs is beyond question, there is very little evidence about factors that affect the acculturation of URMs. But knowing these factors might help to facilitate the acculturation process of URMs and thus, improve the mental health and societal participation of URMs. Some studies have discussed possible factors that influence acculturation. In terms of age, some studies have shown that a younger age might be associated with a stronger orientation toward the host country (Oppedal and Idsoe, 2012, 2015; Lutterbach and Beelmann, 2021). Other studies did not identify any differences in the acculturation strategies in terms of age (Lincoln et al., 2016). Heterogeneous results are also available for gender. Some studies have shown a stronger orientation of refugee girls toward their home country and a weaker orientation toward the host country than of refugee boys (Oppedal and Idsoe, 2012, 2015). Other studies did not identify any gender differences in the acculturation process (Lincoln et al., 2016). Furthermore, several studies have investigated the impact of the time since arrival in the host country on the acculturation process. Overall, these studies suggest that the longer refugees stay in the host country, the stronger the orientations toward the host country would be, whereas the orientation toward the home country diminishes over time (Oppedal and Idsoe, 2015; Lincoln et al., 2016; Jorgenson and Nilsson, 2021; Lutterbach and Beelmann, 2021).

URMs are not only confronted with acculturation tasks in the host country, but they have also to deal with various stress-related factors, such as previous experiences of traumatic events, the asylum process, or the experiences of daily stressors in the receiving society. Nevertheless, studies that investigate the relationship between these stress-related factors and acculturation are rare or lacking completely. Stress symptoms triggered by traumatic experiences such as concentration problems, intrusions, or hyperarousal can make it difficult to learn a new language or build new relationships. Jorgenson and Nilsson (2021) reported in a study of *N* = 80 Somali refugees in the United States that traumatic experiences did not significantly predict the orientation toward the United States. Similarly, for female adult refugees in Germany, Starck et al. (2020) did not identify any significant correlations between the number of traumatic events and the orientation toward the host and home country. However, an association between the asylum process and acculturation should be considered, as factors such as language acquisition or having a job in the host country are both requirements for permanent residence permits (Hornfeck et al., 2022). Furthermore, they are often used as indicators for successful acculturation (El Khoury, 2019; Brook and Ottemöller, 2020). Nevertheless, no empirical evidence on the relationship between the asylum process and acculturation has been provided up to now. Similarly, the relationship between perceived daily stressors and acculturation is still unclear. A study by Safdar et al. (2021) of Syrian refugees in Germany demonstrated a significant positive correlation between daily stressors and host cultural orientation, whereas no significant association was identified between daily stressors and the orientation toward the home country. However, URMs experience a broad spectrum of daily stressor such as social (e.g., difficulties in making friends, conflicts with adults/peers), material (e.g., lack of money, medical care, food), discrimination (e.g., feeling of being treated differently compared to others), or other stressors related to their specific situation (e.g., feeling of insecurity or worries about their documents) (Vervliet et al., 2014). These different daily stressors may have differing effects on the orientation toward the home country and the host country or vice versa.

These stress-related factors can have a severe negative impact on refugee’s mental health (Vervliet et al., 2014; Hornfeck et al., 2022; Pfeiffer et al., 2022). A large body of research has shown, that especially URMs constitute a vulnerable population for developing trauma-related mental health disorder such as posttraumatic stress disorder (PTSD), anxiety or depression (Fazel et al., 2012; Blackmore et al., 2020). To a lesser extent, research has focused on the potentially bi-directional effect of acculturation strategies and mental health state in this population. However, most of the available data are based on cross-sectional study designs, thus the present findings have to be interpreted with caution, and the association must be discussed in both directions (Green et al., 2021). For instance, in a systematic review focusing on migration populations, Choy et al. (2021) had shown that Marginalization was associated with more depression, compared to the other strategies and that those with a marginalized or separated acculturation style showed the highest anxiety-related symptoms. Several studies have shown a similar relation between acculturation and psychosocial wellbeing in URMs (Oppedal and Idsoe, 2012; El-Awad et al., 2021). In general, Assimilation or Integration seem to be stronger associated with improved psychosocial wellbeing, compared to Separation or Marginalization (Oppedal and Idsoe, 2015; Garcia and Birman, 2022). Nevertheless, there are also studies reporting no significant association between acculturation styles and mental health (Copoc, 2019; Green et al., 2021).

It is widely recognized that social support has a beneficial effect on mental health (Oppedal and Idsoe, 2015), and it seems to have a positive impact on the acculturation process, too. Generally speaking, family members may be a great source of social support for adolescents during their acculturation process (Blanc et al., 2022). For URMs, however, this situation is somewhat more complex because their family members do not live with them in their new host country and are not, therefore, in a position to provide the same social support as family members who are present. Oppedal and Idsoe (2015) highlighted the relevance of social support for URMs in Norway with regard to the acculturation process. They reported that social support from family and co-ethnic friends enhanced the orientation toward the home country, and social support from Norwegian friends enhanced the orientation toward the host country.

To summarize, much has still to be learned about the acculturation process of URMs, more precisely which individual level factors (e.g., gender, age), stress-related factors (e.g., traumatic experiences, daily stressors), or contextual factors (e.g., social support) influence the orientation toward the host country or the home country. To our knowledge, no study has examined the potential predictors of the acculturation process of URMs. Moreover, it is still unclear which acculturation strategies are preferred by this heterogeneous population. Consequently, the aim of this analysis was to gain a better understanding of the acculturation process of URMs in Germany. Our expectation was that the most prevalent acculturation strategies of URMs in Germany would be “Integration” and “Assimilation.” This expectation is based on previous acculturation research in Germany with refugees (Copoc, 2019) and the so called “integration measures” the German government is offering to all URMs (e.g., free German language classes; Hertner, 2022) and the accommodation in CWYS, with professional and specifically trained staff, aiming to help the URMs to get used to the new environment.

Furthermore, the investigation aimed to explore the impact of individual characteristics, stress-related and contextual factors on the acculturation process in order to draw conclusions about the implications for further research, and to derive recommendations for political and mental health practice on how to improve the acculturation process of URMs, which might result in improved mental health and societal participation in URMs.

## 2. Methods

### 2.1. Design and procedure

The present study represents a secondary analysis of a subsample of the randomized controlled trial BETTER CARE (bettercare.ku.de; Rosner et al., 2020). The project was approved by the ethics committees at Ulm University (No. 243/19) and at the Catholic University of Eichstätt-Ingolstadt (No. 004-19). CYWS facilities with URMs were contacted through letters of invitation, phone calls, and digital information events. Once a CYWS facility had indicated its willingness to participate in the study, the staff of the CYWS facility identified possible participants, invited and informed them about the study. Inclusion criteria for participants were (1) age 12–20 years, (2) arrived in Germany as unaccompanied minors, (3) applied for asylum or intend to do so, (4) being cared for by a CYWS facility, (5) reported at least one traumatic event in line with the DSM-5 A criterion, and (6) written informed consent given by participant. In the case of minors under the age of 16, their legal guardians were informed and asked for informed written consent.

The assessment took place between July 2020 and October 2021 in the CYWS facilities or in a digital form due to the COVID-19 restrictions. The consent forms and survey measures used in this study were translated by professional translators in cooperation with the study team using the back-translation method (Guillemin et al., 1993) into English, French, Arabic, Dari, Farsi, Pashto, Somali, and Tigrinya.

All variables were assessed using self-report questionnaires on tablets or on paper. Screening appointments were conducted in groups, but the participants filled out the questionnaires by themselves. Each screening appointment took approximately 2 hours for the entire group. Trained staff from the study centers assisted the participants in completing the measures. Interpreters were present when participants were not literate in the languages in which the materials were provided. The participants were given vouchers worth a total of €35 for stores of their choice as compensation for their participation. After the screening, the participants were given a confidential written evaluation of their mental health status along with an individual treatment recommendation.

### 2.2. Measures

#### 2.2.1. Acculturation orientation toward the home country and the host country

Acculturation orientation was assessed using the Brief Acculturation Orientation Scale (BAOS, Demes and Geeraert, 2014). The participants rated eight items (four items each for the orientation toward the home and the host country) on a 7-point Likert scale ranging from 1 (*strongly disagree*) to 7 (*strongly agree*), for instance “In Germany, it is important for me to have friends from my home country.” The BAOS was specifically designed in response to the criticism on Berry’s model (Maehler and Shajek, 2016), by using continuous variables to measure acculturation instead of dichotomous variables. Previous studies showed good reliability for adult refugees (α = 0.87–0.89; Safdar et al., 2021). In the present study, both subscales showed good reliability (Cronbach’s α_BAOS-Home_ = 0.80, Cronbach’s α_BAOS-Host_ = 0.76).

#### 2.2.2. Daily stressors

The experience of post-migration daily stressors was assessed using the Daily Stressors Scale for Young Refugees (DSSYR, Vervliet et al., 2014). On a 4-point Likert scale from 0 (*not*) to 3 (*very much*) the participants rated the extent to which they had experienced 19 different daily stressors, for instance “not enough food” (possible range: 0–57). The reliability of this scale was good (α = 0.86). This questionnaire is widely used in research with URMs (α = 0.79–0.91; Behrendt et al., 2022; Spaas et al., 2022). It was developed on the basis of the Columbia Impairment Scale (CIS), the Adolescents Complex Daily Stressors Scale (ACDSS), and the authors’ own experiences in the field. Nevertheless, the validation study of this measure has not yet been published.

#### 2.2.3. Traumatic experiences

The Child and Adolescent Trauma Screen was used to assess the number of traumatic events experienced (CATS-2, Sachser et al., 2022). The event checklist with 15 possible traumatic events was presented, for instance “Threatened, hit or hurt badly in my family.” Participants could answer “*yes*” or “*no*” to indicate whether they had experienced the presented traumatic event or not. Previous studies with URMs (Müller et al., 2019; Pfeiffer et al., 2022) have shown sufficient to good reliabilities (α = 0.75–0.83). The reliability in the present study was excellent (α = 0.92).

#### 2.2.4. Social support

Social support was assessed using an adapted version of the Social Support Questionnaire (SSQ6-G; Leppin et al., 1986). The general question “To whom can you turn confidentially, if you are in trouble or have problems, if you are in a bad mood, afraid or oppressed?” was asked and the participants could indicate the amount of support they receive from different contact persons (e.g., siblings, teacher) from *1* (*most likely*) to *4* (*never*) (13 items, α = 0.74). In the present investigation, the items of the SSQ6-G were dichotomized into *yes* (answers *1–3*) and *no* (answer *4*), and summed up to report the number of possible contact persons. In its original version, the SSQ6-G showed good reliability (α = 0.71 to 0.92).

Furthermore, contact with their families was assessed by means of one item “Do you have contact to your family” and a 6-point Likert scale [0 (*No*), 1 (*Yes, once a year or less*), 2 (*Yes, several times a year*), 3 (*Yes, at least once a month*), 4 (*Yes, at least once per week*), and 5 (*Yes, daily*)].

#### 2.2.5. Group climate

The Group Climate in the CYWS was measured by the Group Climate Instrument for Children (Strijbosch et al., 2014). It contains 14 items and two subscales: open climate (9 items, α = 0.94) and closed climate (5 items, α = 0.50). Participants rated the items on a 5-point Likert-scale from 1 (*I do not agree*) to 5 (*I totally agree*). Due to the low reliability, this questionnaire was excluded in the present analysis.

### 2.3. Statistical analysis

Data analyses were conducted using IBM SPSS Statistics for Windows, Version 28.0. To profile the sociodemographic, the stress-related, and the contextual factors of the sample, descriptive statistics (means, standard deviation, ranges, and frequencies) and correlations were computed. To explore the frequencies of the four different acculturation strategies, the subscales of the BAOS were dichotomized at the scale center, and the participants were assigned to clusters according to how their orientation toward their home country and toward their host country Germany was described. In response to criticism of Berry’s dimensional model of acculturation (Schwartz et al., 2010), the variables of the orientation toward the home country and the host country were deemed to be continuous in all further analyses in this study. Correlation analysis and regression models were calculated separately for each variable. For the DSSYR, a sum score of all items was calculated to capture both the number and the experienced intensity of daily stressors. For the CATS-2, the number of traumatic events were included, using the sum score of the CATS-2 event checklist.

Two multiple linear hierarchical regression analyses were conducted to examine the relative contribution of individual factors, as well as stress-related and contextual factors to the orientation toward the home country and the orientation toward the host country, separately. For each hierarchical regression, the first model included individual demographic characteristics, such as age, gender, and length of stay in the host country. The second model included stress-related factors, such as the number of traumatic experiences, the daily stressors, and the stress caused by asylum status. A third model included contextual factors such as social support. The dependent variable in the regressions was either the mean score of the BAOS subscale “orientation toward the home country” or “orientation toward the host country.”

In a second step, the items of the DSSYR were entered as individual items instead of the sum score in order to consider the heterogeneity of the different types of daily stressors, and to investigate their individual impact. Consequently, the correlation between the single variables of the DSSYR and the BAOS subscales was calculated, and two additional multiple regression models were calculated. The first regression analysis targeted the orientation toward the home country and the second regression analysis the orientation toward the host country, including all 19 items of the DSSYR as independent variables.

A level of significance of *p* < 0.05 (two tailed) was predetermined in all analyses. Due to the exploratory character of the present analysis, no multiple test adjustments were necessary (Bender and Lange, 2001).

## 3. Results

Altogether *N* = 132 URMs who lived in 22 different CYWS facilities in Germany were included in the present investigation. Eight participants were excluded because of missing data (more than 30% of the items in one questionnaire were unanswered). Of the participants, *n* = 101 (82.5%) identified themselves as male, *n* = 22 (17.7%) as female, and *n* = 1 (0.8%) as diverse. Hence, the final sample for this investigation consisted of *N* = 124 URMs. They ranged in age between 13 and 20 (*M* = 16.94; *SD* = 1.47). They were born in 28 different countries, mainly in the Middle East (e.g., Afghanistan *n* = 38; Syria *n* = 12) and in African countries (e.g., Somalia *n* = 15; see Appendix for a detailed list). The length of stay in Germany ranged from 1 to 90 months (*M* = 25.20, *SD* = 20.40). Table 1 contains the descriptive characteristics of the full study sample (*N* = 124) and intercorrelations for each study variable. The participants reported between 1 and 14 traumatic events (*M* = 6.57, *SD* = 3.07). Furthermore, *n* = 43 (34.7%) had no contact with their family, *n* = 31 (25.0%) reported having contact with their family at least once a week, and *n* = 19 (15.3%) had contact with their family on a daily basis.

**Table 1:** Descriptive statistics and correlations for study variables.

Variable	*M* or *n*	*SD* or %	1	2	3	4	5	6	7a	7b	8
1. Gender (male)	101	82.1									
2. Age	16.94	1.47	0.07	–							
3. Length of stay in Germany	25.20	20.40	−0.02	0.30^**^	–						
4. Traumatic events	6.57	3.07	0.07	0.07	0.05	–					
*5.* Daily stressors	16.27	9.08	−0.01	−0.03	−0.11	0.34^**^	–				
6. Stress because of asylum status	5.59	3.35	0.00	0.00	−0.22^*^	0.36^**^	0.27^**^	–			
*Social Support*											
7a. Social support	5.89	2.46	−0.07	0.10	0.14	−0.06	0.10	−0.13	–		
7b. Contact with family	2.31	1.97	−0.16	0.01	0.31^**^	−0.20^*^	−0.07	−0.27^**^	0.16	–	
*Acculturation Orientations*											
8. Orientation home country	4.09	1.60	−0.02	−0.07	0.05	−0.14	0.20^*^	−0.04	0.08	0.10	–
9. Orientation host country	5.36	1.26	−0.04	−0.01	−0.14	0.09	0.12	0.26^**^	−0.06	−0.12	0.19^*^

Values are means ± SD or *n* (%), as appropriate.

^*^p < 0.05. ^**^p < 0.01.

### 3.1. Current acculturation patterns of participating URMs

**Figure 1:**
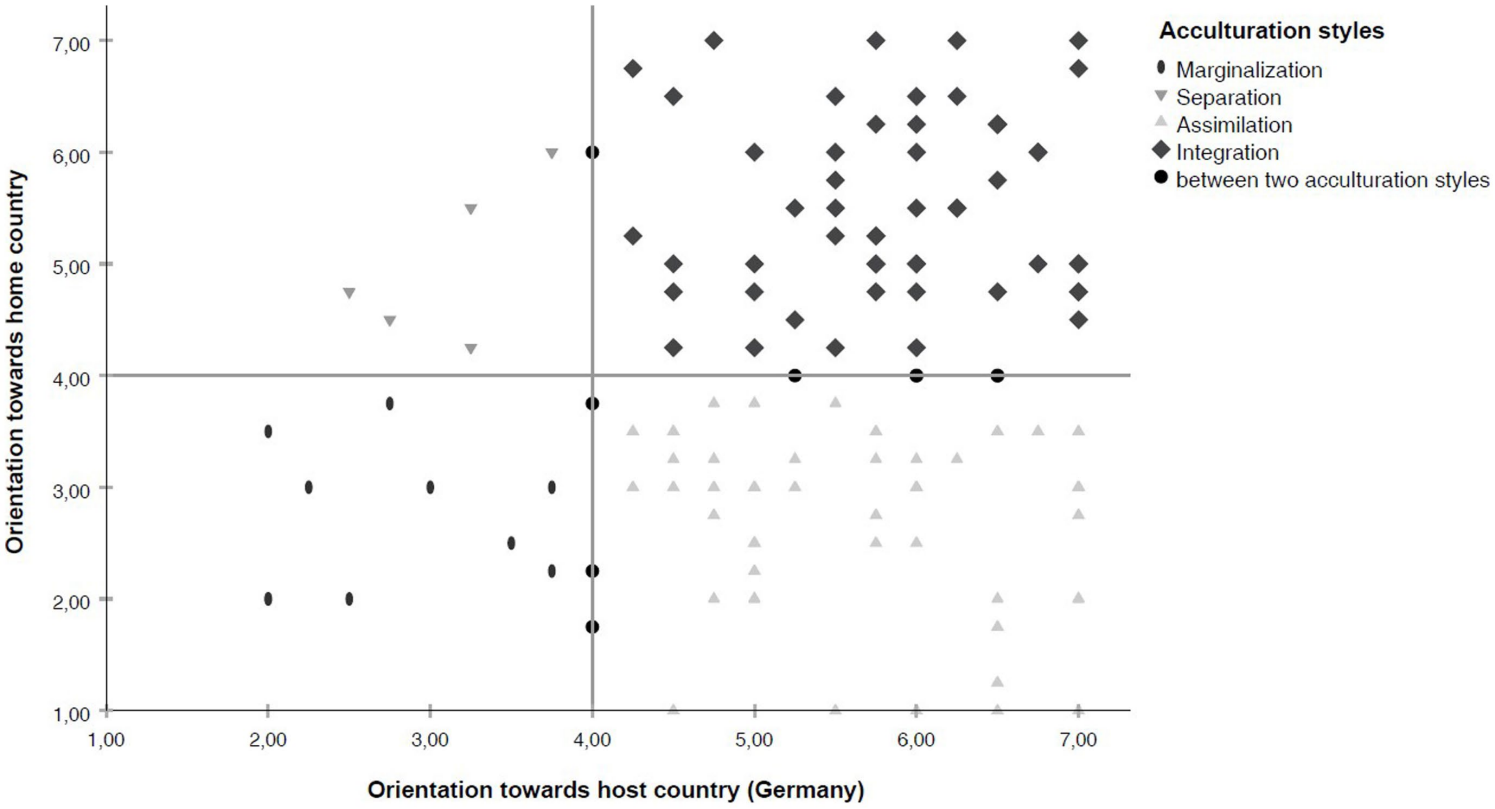
Acculturation styles.

The participants scored higher than the mid-point on the scales of the orientation toward the home country (*M* = 4.09, *SD* = 1.60) and the orientation toward the host country Germany (*M* = 5.36, *SD* = 1.26). Of the participants, *n* = 10 (8.1%) displayed a marginalized, *n* = 5 (4.0%) a separated, *n* = 46 (37.1%) an assimilated, and *n* = 54 (43.5%) an integrated acculturation style. The values of *n* = 9 (7.3%) participants were positioned between two acculturation styles. For more details, see Figure 1.

### 3.2. Contribution of individual, stress-related, and contextual factors to the acculturation process

The results indicated that the orientation toward the host country Germany correlated positively with the reported stress regarding asylum status (*r* = 0.261, *p* = 0.003, 95% CI [0.089; 0.418]), and correlated positively with the orientation toward the home country (*r* = 0.190, *p* = 0.035, 95% CI [0.014; 0.354]). Furthermore, the orientation toward the home country correlated positively to a significant degree with the experience of daily stressors (*r* = 0.201, *p* = 0.025, 95% CI [0.026; 0.364]). None of the other variables correlated significantly with the acculturation scales.

For the dependent variable orientation toward the home country, only the second model was significant [*R*^2^ = 0.121, *F*(6, 115) = 2.648; *p* = 0.019]. The results of the hierarchical regressions are presented in Table 2. The number of traumatic events was associated negatively with the orientation toward the home country (β = −0.264, *p* = 0.009), while the experience of daily stressors was associated positively with the orientation toward the home country (β = 0.310, *p* = 0.001). Model 3 was not significant. No regression model for the dependent variable of orientation toward the host country was significant (see Table 3).

**Table 2:** Hierarchical regression with orientation toward the home country as dependent variable.

Variable	*B*	95% *CI* for *B*		*SE B*	β	*R* ^2^	*ΔR* ^2^
		LL	UL				
Step 1						0.014	0.014
Constant	5.94^***^	2.57	9.32	1.71			
Age	−0.12	−0.32	0.09	0.10	−0.11		
Gender	0.25	−0.50	1.00	0.38	0.06		
Length of stay	0.01^*^	−0.01	0.02	0.01	0.06		
Step 2						0.121^*^	0.107^***^
Constant	5.71^***^	2.43	8.99	1.66			
Age	−0.11	−0.30	0.09	0.10	−0.10		
Gender	0.36	−0.36	1.08	0.36	0.09		
Length of stay	0.01	−0.01	0.02	0.01	0.10		
Traumatic events	−0.14^**^	−0.24	−0.04	0.05	−0.26		
Daily stressors	0.05^***^	0.02	0.09	0.02	0.31		
Asylum stress	−0.01	−0.10	0.09	0.05	−0.01		
Step 3						0.124	0.003
Constant	5.51^**^	2.11	8.90	1.71			
Age	−0.11	−0.31	0.09	0.10	−0.10		
Gender	0.40	−0.34	1.13	0.37	0.10		
Length of stay	0.01	−0.01	0.02	0.01	0.09		
Traumatic events	−0.13^*^	−0.24	−0.03	0.05	−0.26		
Daily stressors	0.05^**^	0.02	0.09	0.02	0.30		
Asylum stress	−0.00	−0.10	0.09	0.05	−0.00		
Social support	−0.03	−0.09	0.15	0.06	0.05		
Contact with family	0.02	−0.13	0.18	0.08	0.03		

CI, confidence interval; LL, lower limit; UL, upper limit. *N* = 123.

^*^p < 0.05. ^**^p < 0.01. ^***^p < 0.001.

**Table 3:** Hierarchical regression with orientation toward the host country as dependent variable.

Variable	*B*	95% *CI* for *B*		*SE B*	β	*R* ^2^	*ΔR* ^2^
		LL	UL				
Step 1						0.021	0.021
Constant	20.28^***^	9.55	31.01	5.42			
Age	0.13	−0.52	0.78	0.33	0.04		
Gender	−0.18	−2.56	2.20	1.20	−0.01		
Length of stay	−0.04	−0.09	0.01	0.02	−0.15		
Step 2						0.077	0.056
Constant	18.55^***^	7.82	29.28	5.42			
Age	0.07	−0.57	0.71	0.32	0.02		
Gender	−0.23	−2.59	2.12	1.19	−0.02		
Length of stay	−0.02	−0.07	0.03	0.02	−0.09		
Traumatic events	0.00	−0.33	0.33	0.17	0.00		
Daily stressors	0.02	0.09	0.13	0.05	0.04		
Asylum stress	0.34^*^	−0.05	0.64	0.15	0.23		
Step 3						0.079	0.002
Constant	19.10^***^	8.01	30.20	5.60			
Age	0.07	−0.58	0.72	0.33	0.02		
Gender	−0.32	−2.72	2.09	1.22	−0.02		
Length of stay	−0.02	−0.07	0.03	0.03	−0.08		
Traumatic events	−0.01	−0.35	0.33	0.17	0.01		
Daily stressors	0.03	−0.08	0.14	0.06	0.05		
Asylum stress	0.33^*^	0.03	0.64	0.16	0.22		
Social support	−0.03	−0.42	0.36	0.19	−0.02		
Contact with family	−0.11	−0.62	0.41	0.26	−0.04		

CI, confidence interval; LL, lower limit; UL, upper limit. *N* = 123.

^*^p < 0.05. ^**^p < 0.01. ^***^p < 0.001.

### 3.3. Contribution of daily stressors to the acculturation process

Table 4 shows the correlations between the items of the DSSYR and the orientation toward the host and the home countries. Difficulties in obtaining official documents were related positively to a significant degree with the orientation toward Germany (*r* = 0.184, *p* = 0.050; 95% CI [0.000; 0.355]). Not having enough food (*r* = 0.268, *p* = 0.003; 95% CI [0.092; 0.427]), clothes (*r* = 0.269, *p* = 0.003; 95% CI [0.096; 0.427]), and/or money (*r* = 0.376, *p* < 0.001; 95% CI [0.211; 0.520]) as well as involuntarily changes to the CYWS facility (*r* = 0.199, *p* = 0.029, 95% CI [0.021; 0.365]) correlated positively with the orientation toward the home country. Difficulties in finding new friends in Germany correlated negatively with the orientation toward the home country (*r* = −0.215, *p* = 0.017; 95% CI [−0.377; −0.039]). Further multiple linear regression analyses were conducted to examine the relative contribution of the items of the DSSYR. The regression model for the dependent variable of orientation toward the host country Germany was not significant [*R*^2^ = 0.22, *F*(19, 70) = 1.01, *p* = 0.457], but the regression model for the dependent variable of orientation toward the home country was significant [*R*^2^ = 0.34, *F*(19, 70) = 1.88, *p* = 0.03; see Table 5]. The lack of money (β = 0.377, *p* = 0.019, 95% CI [0.096; 1.034]) and difficulties in making new friends (β = −0.284, *p* = 0.014; 95% CI [−0.914; −0.104]) was associated with the orientation toward the home country.

**Table 4:** Pearson correlation of items of DSSYR and BAOS Subscales.

	*n*	*M*	*SD*	BAOS-Host (*r*)	BAOS-Home (*r*)
Insufficient food	119	0.54	0.87	0.141	0.268^**^
Insufficient clothes	122	0.92	1.12	0.110	0.269^**^
Insufficient money	120	1.40	1.02	0.091	0.376^***^
Insufficient housing	122	0.47	0.86	0.135	0.175
Insufficient medical care	122	0.52	0.90	0.098	0.069
Insufficient access to education	122	0.51	0.87	0.066	0.178
Lack of information (about my rights, ongoing proceedings)	120	0.82	0.90	0.155	0.106
Feelings of insecurity	124	0.69	0.98	0.037	−0.033
Difficulties in making friends	123	0.73	0.94	0.012	−0.215^*^
Worries about family at home	112	2.13	1.02	0.031	0.115
Difficulties in obtaining legal residence documents	115	1.54	1.21	0.184^*^	0.024
Problems related to the age assessment procedure	115	0.41	0.83	−0.008	0.064
Difficulty communicating with others in the foreign language	124	1.11	0.97	0.128	0.146
Multiple involuntary changes of accommodation	121	0.68	1.05	0.148	0.199^*^
Boredom	124	1.21	0.94	−0.138	0.079
Feeling uncertain about the future	121	1.30	0.99	0.117	−0.028
Hearing people say bad things about myself	114	0.59	0.75	−0.116	−0.004
Feeling of being threatened differently compared to others	121	0.56	0.77	−0.085	0.005
Feeling that others have prejudices about myself or people of my country/culture	120	0.78	0.84	−0.066	−0.084

DSSYR, daily stressors scale for young refugees; BAOS, brief acculturation orientation scale.

^*^p < 0.05. ^**^p < 0.01. ^***^p < 0.001.

**Table 5:** Multiple linear regressions for orientation toward the home country.

Variable	B	SE B	95% CI for B		β	*t*	*p*
			LL	UL			
Insufficient food	0.28	0.24	−0.21	0.77	0.16	1.15	0.254
Insufficient clothes	0.06	0.23	−0.41	0.52	0.04	0.25	0.804
Insufficient money	0.57^*^	0.24	0.10	1.03	0.38	2.40	0.019
Insufficient housing	0.09	0.34	−0.59	0.76	0.04	0.26	0.798
Insufficient medical care	−0.04	0.30	−0.63	0.55	−0.02	−0.13	0.896
Insufficient access to education	−0.11	0.30	−0.71	0.49	−0.06	−0.36	0.721
Lack of information (about my rights, ongoing proceedings)	0.36	0.24	−0.11	0.83	0.20	1.53	0.131
Feelings of insecurity	0.34	0.29	−0.23	0.91	0.17	1.18	0.241
Difficulties in making friends	−0.51^*^	0.20	−0.91	−0.10	−0.28	−2.51	0.014
Worries about family at home	0.14	0.18	−0.23	0.50	0.09	0.74	0.461
Difficulties in obtaining legal residence documents	−0.06	0.16	−0.38	0.27	−0.04	−0.35	0.726
Problems related to the age assessment procedure	−0.08	0.28	−0.63	0.47	−0.04	−0.30	0.768
Difficulty communicating with others in the foreign language	0.23	0.18	−0.13	0.60	0.15	1.29	0.201
Multiple involuntary changes of accommodation	0.09	0.22	−0.36	0.53	0.06	0.40	0.688
Boredom	0.03	0.21	−0.38	0.44	0.02	0.14	0.889
Feeling uncertain about the future	−0.13	0.23	−0.58	0.32	−0.08	−0.56	0.575
Hearing people say bad things about myself	−0.17	0.28	−0.73	0.39	−0.08	−0.60	0.554
Feeling of being threatened differently compared to others	−0.39	0.28	−0.95	0.18	−0.19	−1.37	0.174
Feeling that others have prejudices about myself or people of my country/culture	−0.30	0.26	−0.82	0.21	−0.16	−1.17	0.246

*R*^2^ = 0.337; *F*(19, 70) = 1.876; *p* = 0.030. ^*^p < 0.05.

## 4. Discussion

The current study investigated the prevalence of acculturation strategies of URMs in Germany and possible predictors for orientation toward the host country and the home country. As expected, Integration (43.5%) and Assimilation (37.1%) were the most frequent acculturation styles reported by the participants. This is in line with previous studies with adult refugees in Germany (Copoc, 2019). It may back the assumption that the attitudes of the host society play a major role in the acculturation process of refugees (Berry, 1997). In the case of Germany, previous research has shown that the majority population expected refugees to assimilate into German society (Hertner, 2022). URMs in Germany might be confronted with these expectations through overt or subtle comments, behaviors, or policies (Katbeh, 2020), and react consciously or unconsciously with a stronger orientation toward the host country. This assumption is also supported by our finding that none of the studied factors were associated with the orientation toward the host country. This suggest that the attitudes of the receiving society might play an important part in the acculturation process of URMs. Nevertheless, other factors, such as the language proficiency of the URMs (Beißert et al., 2020) or the cultural distance between the home country and the host country (Sam and Berry, 2006) were not included in the present investigation, but might impact the acculturation process of URMs.

Similar to previous studies (Müller et al., 2019), the participants reported having experienced a high number of traumatic events in the present study, too. Higher numbers of traumatic events experienced by the URMs were linked to weaker orientation reported toward their home country or vice versa. It is common knowledge that URMs frequently experienced traumatic events in their home country (Pfeiffer et al., 2022). The weaker orientation toward the home country in relation to experiences of traumatic events might, therefore, constitute a coping strategy of the URMs to avoid being reminded of these traumatic experiences. Nevertheless, our findings contrasted with those of Jorgenson and Nilsson (2021). They did not report any significant association between traumatic experiences and acculturation. Jorgenson and Nilsson (2021) focused on adult Somali refugees in the United States whereas the participants in the present study were URMs in Germany. These two samples differ significantly regarding age and country of origin, and also regarding their migration journey, their current accommodation situation, and their current societal circumstances. Since individual, familial, and contextual factors should be considered when discussing the psychosocial well-being and the acculturation process of refugees (Demes and Geeraert, 2014; Arakelyan and Ager, 2021), generalizations between different refugee populations should be avoided. Instead, our investigation highlights the importance of taking a differentiated look at URMs. It suggests that the traumatic experiences of URMs in Germany might have a relation to the acculturation process that might be different from that of adult refugees.

Furthermore, the URMs reported a high number of daily stressors. This is in line with previous research with URMs after resettlement (Pfeiffer et al., 2022), and highlights the challenging living situation of URMs. In the present investigation, the experience of daily stressors was associated with a stronger orientation toward the home country. This means, that URMs who experienced more daily stressors might show a stronger orientation toward their home country or vice versa. This outcome contrasted with Safdar et al. (2021) who did not report any association between daily stressors and the orientation toward the home country but suggested an association between daily stressors and the orientation toward the host country. There are several possible explanations for this result. The study by Safdar et al. (2021) was conducted with adult Syrian refugees and not with URMs. Moreover, to measure the perceived daily stressors, they used a checklist developed for Vietnamese immigrants in Canada (Lay and Nguyen, 1998) and not a dedicated instrument to measure the daily stressors young refugees may perceive in the host country like the one used in the present study. Nevertheless, it has to be acknowledged that URMs do experience specific daily stressors (Vervliet et al., 2014). It is, therefore, necessary to use special instruments to accommodate this situation. Hence, the present investigation suggests that, for URMs, the experience of daily stressors might lead to a stronger orientation toward their home country, potentially as a way of coping with negative experiences in the host country.

When considering the effects of different stressors independently, our results suggest that, more particularly, the perceived lack of money and fewer difficulties in making new friends might be related with the orientation toward the home country. Vervliet et al. (2015) investigated the aspirations of Afghan refugee minors in Belgium. In this host country, 67% of the participants had the aspiration of earning money for themselves and 56% had the aspiration of earning money for their family. In Germany, while living in CYWS facilities, the URMs only receive a small amount of pocket money according to German law (§34 SBG VIII). Therefore, these aspirations often cannot be fulfilled (Thomas et al., 2018). This might be linked to homesickness (Rosner et al., 2022). The feeling of homesickness might subsequently be related to URMs thinking more about their home country and might be correlated to the orientation toward the home country—as demonstrated in this investigation.

In the present investigation, difficulties in making new friends were negatively associated with the orientation toward the home country. Participants who faced more difficulties in making new friends reported a weaker orientation toward their home country, or vice versa. No significant association with the orientation toward the host country was shown. At first glance, this finding may seem counterintuitive as social support by peers has been identified as an important factor in the acculturation process (Oppedal and Idsoe, 2015). Nevertheless, previous research differentiated between the effect of having friends in the host country and having co-ethnic friends. On the one hand, having friends from the host country might lead to an increase of the orientation toward the host country—by learning the new language or coming into contact with traditions of the host country. On the other hand, having co-ethnic friends might lead to an increase of the orientation toward the home country by offering the possibility of integrating traditions or habits from their home country into their new living environment (Oppedal and Idsoe, 2015; Behrendt et al., 2021). Consequently, the reported difficulties in making new friends might be linked to the wish of having more co-ethnic friends in the host country. This might, in turn, lead to an increased orientation toward the host country.

### 4.1. Limitations

Several limitations to this investigation have to be borne in mind. First, acculturation must be understood as a lifelong process of negotiation between the home culture and the host culture (Sam and Berry, 2006). Moreover, the participants in the present study came from very different countries of origin and therefore, might have encountered very different acculturation challenges, depending on the customs in their home country. The results can, therefore, only give a first overview about the acculturation of URMs but cannot be generalized for all URMs. Moreover, given the high proportion of male participants (82.5%), the results cannot be easily transferred to merely female groups. Future studies should attempt to work with a larger sample size in order to provide more differentiated views of the acculturation process of URMs.

Second, acculturation is a complex process and might take place differently in various spheres of life (e.g., private vs. public, school vs. home; Safak-Ayvazoglu and Kunuroglu, 2021). This factor also needs to be discussed against the backdrop of the attitudes of the host country (Sam and Berry, 2006). The results of this investigation give a first impression of the overall acculturation orientations of URMs in Germany. Further studies should differentiate between various spheres, and also examine the attitudes of the members of the host country. Qualitative measures could provide further insights, and should be used in future studies.

Third, the results are only based on cross-sectional data, not allowing any causal one-directional conclusions. Future studies should investigate these potentially bi-directional effects in longitudinal study designs to gain further insights into the acculturation process of URMs. Nevertheless, URMs are a very mobile sample (Keles et al., 2018), and longitudinal studies with this population are, therefore, quite challenging, leading to a lack of follow-up data.

Fourth, in respect to the used questionnaires, the CATS-2 is only using a standardized checklist of potentially traumatic events, thus we cannot draw any conclusions about subjective experience behind the reported events. Furthermore, due to the unreliability of the Group Climate Instrument for Children (Strijbosch et al., 2014), this instrument could not be included in the analysis. Consequently, a limited number of contextual factors were considered in the present study. However, the acculturation process is also significantly influenced by societal and contextual factors, and should not be seen as the responsibility of the refugees alone. Further acculturation studies with URMs should take this aspect into account, especially as, in the present investigation, many individual factors did not play a significant role in the acculturation process of URMs.

Sixth, the present investigation was a secondary analysis of the randomized controlled trial “BETTER CARE” (Rosner et al., 2020). Therefore, there was no a priori power calculation made for this specific analysis. Moreover, due to the exploratory character of the present investigation, no alpha correction was made (Bender and Lange, 2001). Nevertheless, there is a potential risk of alpha error inflation, thus generalizations of the present findings should be avoided and previous studies are needed to replicate our findings.

### 4.2. Implications

Based on the research findings, several implications and recommendations could be derived that could help to improve the successful acculturation process of URMs in Germany. The beneficial acculturation patterns displayed by the URMs highlighted the positive effects of the German so-called “integration measures” (language courses, integration classes, accommodation in CYWS) for URMs. Nevertheless, there is a need to focus more on those who have not yet benefited from these offers, and who potentially displayed less favorable acculturation patterns such as Separation and Marginalization. Having identified specific factors that might have an influence on the acculturation process of URMs, the mental health of this vulnerable population can be improved by clinical, pedagogical and policy interventions that target the individual acculturation process more thoroughly. One way of doing this would be for policymakers and practitioners to focus not only on the orientation toward the host country, but also to consider the orientation toward the home country in their interventions and measures. Up to now, so-called “integration measures” in Germany have mainly sought to strengthen the orientation toward the host country, for example, by offering language courses (Katbeh, 2020; Hertner, 2022). Nevertheless, the present investigation highlights the relevance of the orientation toward the home country when dealing with negative experiences in the host society. Moreover, the association between difficulties in making new friends and poorer orientation toward the home country highlights the importance of creating opportunities for interaction and making friends with both co-ethnic peers and peers from the host society. Therefore, it is crucial to offer acculturation-based programs that strengthen both the orientation toward the home and the host country. This would give URMs an opportunity to develop their own bi- or multicultural identity and to acculturate according to their needs and at their own pace. In the long term, such changes, on the practical and political level, could impact the societal discourse in Germany leading to a shift from the expectation of assimilation (Katbeh, 2020) toward the “real” integration of minority groups such as URMs into the majority society. Moreover, as acculturation always depends on the host society, interventions should not refer solely to URMs, but also consider the perspective, expectations, and participation of the majority society in order to generate a holistic view of the “integration discourse.”

### 4.3. Conclusion

In sum, URMs in Germany mainly showed favorable acculturation patterns. Stress-related factors such as traumatic experiences and daily stressors may impact the acculturation process of URMs. The investigation highlights possible improvements in policy and practice, which could have lasting positive effects not only on the psychosocial health and social participation of URMs, but also on society as a whole. Future research should investigate the acculturation process in a longitudinal design, possibly over several years and across different developmental phases of the young refugees.

## Data availability statement

The datasets generated for this study are available from the corresponding author on request.

## Ethics statement

The studies involving human participants were reviewed and approved by ethics committees at Ulm University (No. 243/19) and at the Catholic University of Eichstätt-Ingolstadt (No. 004-19). Written informed consent to participate in this study was provided by the participants and their legal guardians if necessary.

## Author contributions

HK, RR, CS, and EP designed the study and were responsible for securing the funding. MG, JE, CS, and EP collected the data. MG performed the statistical analysis and drafted the paper under the supervision of EP and CS. All authors contributed to the article and approved the submitted version.

## Funding

This work is part of the BETTER CARE project supported by the German Ministry of Education and Research (01EF1802A, 01EF1802B, and 01EF1802C).

## Conflict of interest

The authors declare that the research was conducted in the absence of any commercial or financial relationships that could be construed as a potential conflict of interest.

## Publisher’s note

All claims expressed in this article are solely those of the authors and do not necessarily represent those of their affiliated organizations, or those of the publisher, the editors and the reviewers. Any product that may be evaluated in this article, or claim that may be made by its manufacturer, is not guaranteed or endorsed by the publisher.
