# Supplementary material for: Factors affecting the acculturation strategies of unaccompanied refugee minors in Germany
Source: Front Psychol. 2023 Jun 19;14:1149437. doi: 10.3389/fpsyg.2023.1149437 (PMC10315911; doi:10.3389/fpsyg.2023.1149437)
Supplement: Supplementary file 1 [file Table_1.DOCX]

**Appendix A**

*Countries of origin of the sample*

| Country of origin | *n* | % |
| --- | --- | --- |
| Afghanistan | 38 | 30.6 |
| Somalia | 15 | 12.1 |
| Syria | 12 | 9.7 |
| Guinea | 7 | 5.6 |
| Iraq | 7 | 5.6 |
| Iran | 7 | 5.6 |
| Sierra Leone | 5 | 4.0 |
| Gambia | 4 | 3.2 |
| Mali | 4 | 3.2 |
| Pakistan | 4 | 3.2 |
| Ethiopia | 2 | 1.6 |
| Eritrea | 2 | 1.6 |
| Turkey | 2 | 1.6 |
| Albania | 1 | 0.8 |
| Algeria | 1 | 0.8 |
| Angola | 1 | 0.8 |
| Azerbaijan | 1 | 0.8 |
| Bangladesh | 1 | 0.8 |
| Benin | 1 | 0.8 |
| Ivory Coast | 1 | 0.8 |
| Kameron | 1 | 0.8 |
| Kenia | 1 | 0.8 |
| Mongolia | 1 | 0.8 |
| Nigeria | 1 | 0.8 |
| Rumania | 1 | 0.8 |
| Sudan | 1 | 0.8 |
| Tunisia | 1 | 0.8 |
| Vietnam | 1 | 0.8 |

*Note*. *N*=123 Unaccompanied Refugee Minors.
